# Effects of Dietary Garlic Skin Based on Metabolomics Analysis in the Meat Quality of Black Goats

**DOI:** 10.3390/foods14111911

**Published:** 2025-05-28

**Authors:** Wanyi Zeng, Xiaoyun Shen

**Affiliations:** College of Life Science and Agri-forestry, Southwest University of Science and Technology, Mianyang 621010, China; zengwy1118@163.com

**Keywords:** meat quality, black goat, garlic skin, metabolomics

## Abstract

This study investigated the potential of dietary garlic skin supplementation to enhance meat quality in black goats, addressing the growing demand for natural feed additives in sustainable livestock production. Twelve 4-month-old Youzhou black goats were randomly assigned to a control group (FR, basal diet) or an experimental group (NR, 16% garlic skin supplementation) for 90 days. Meat quality parameters, amino acid and fatty acid profiles, and metabolomic pathways were systematically analyzed. The NR group demonstrated significantly reduced backfat thickness (*p* < 0.05), increased eye muscle area (*p* < 0.05), higher pH at 0 h post-slaughter (*p* < 0.05), and improved meat color (L* and a* values at 24 h, *p* < 0.05) compared to FR. Cooking loss was significantly lower in NR (*p* < 0.05), alongside elevated arginine and n-3 PUFA (α-linolenic acid, EPA, DHA) levels (*p* < 0.01) and a favorable n-6/n-3 ratio. Metabolomics identified 1970 differentially abundant metabolites, with key enrichments in propionate metabolism, oxidative phosphorylation, and amino acid pathways, notably linking acylcarnitines to color stability and water retention. These findings indicated that garlic skin improved meat quality through coordinated regulation of lipid metabolism, antioxidant enhancement, and mitochondrial optimization. The study provided foundational evidence for utilizing garlic byproducts as functional feed additives to improve nutritional and sensory attributes in goat meat, supporting sustainable meat production strategies.

## 1. Introduction

Goat meat is regarded as a delicious and nutritious choice, vital for human health, owing to its substantial presence of indispensable amino acids and micronutrients, as well as its low calorie and cholesterol content [1]. Meanwhile, black goats, characterized by their entirely black coats, exhibit strong adaptability, cold resistance, disease resistance, robust foraging abilities, tolerance to coarse feed, tender meat texture, high protein content, and low-fat levels. Compared to other local white goats, they are distinguished by their superior meat quality [2]. The black goat not only offers abundant nutrition for consumers but is also highly favored by breeders due to its ease of rearing.

The production of goat meat has expanded to meet rising consumer demand for grass-fed and organically produced meats, as well as processed meat innovations like artisanal sausages, gourmet burgers, and meatballs [3]. This trend corresponds to the evolution of the global economy, where goat meat quality has garnered increasing attention from both the livestock industry and consumers [4]. Therefore, enhancing the sensory and nutritional attributes of goat meat has emerged as a priority of research. Recent studies highlighted multiple determinants influencing meat quality, such as genetic variability (species, age, gender, genotype), dietary composition, supplementary additives, grazing management, and postmortem preservation protocols [5,6]. Thus, optimizing these determinants is essential to drive goat meat industry advancement, enhancing both nutritional value and market competitiveness.

Garlic (*Allium sativum*), a widely utilized culinary ingredient, demonstrates a global annual production exceeding 20 million metric tons [7]. Current research confirms that garlic and its bioactive constituents modulate rumen fermentation patterns and enhance growth performance in ruminants [8]. However, as a secondary product resulting from the processing of garlic, the role of garlic skin is often underestimated. In fact, garlic skin contains analogous antibacterial and antioxidant phytochemicals similar to those in garlic bulbs, such as alliin [9]. Yuan et al. found that supplementing allicin (0.75 g per head per day) could significantly improve the feed intake, growth performance, slaughter performance, antioxidant, anti-inflammatory, immunomodulatory and economic benefits of Guizhou black goats, and also had a hypoglycemic effect [10]. Coupled with low moisture content and active compounds, garlic skin may optimize the fermentation quality of high-moisture silage [11]. Garlic’s demonstrated antibacterial activity has prompted scientific evaluation of its efficacy as a phytogenic growth enhancer in monogastric livestock systems, particularly poultry and chickens [12]. In addition, Zhu et al.’s research found that over a 56-day period, adding 80 g/kg (DM basis) of garlic skins could increase the average daily gain (ADG) and feed conversion rate (FCR) of Hu lambs [8]. Xu et al. found that feeding garlic peels, which account for 8% of the daily diet, can regulate protein synthesis and energy metabolism in fattening lambs, improve the composition of the gut intestinal microbiota, enhance serum antioxidant capacity, boost the body’s immunity, and improve the growth performance and health level of fattening lambs [13]. The utilization of agricultural by-products (such as garlic waste and garlic peels) as animal feed and functional feed additives is crucial for the effective management of organic resources in China. This not only increases the self-sufficiency rate of food and reduces the reliance on imported feed, but also promotes plant-based supplementation strategies aimed at producing high-quality meat.

Based on the above content, this study posits that controlled dietary addition of garlic skin inclusion (16% DM basis) in black goats would enhance meat quality. To validate this hypothesis, a non-targeted metabolomics approach using UHPLC-QTOF-MS was employed to investigate the metabolic pathways regulated by garlic skin supplementation and identify candidate biomarkers associated with meat quality.

## 2. Materials and Methods

Black goats were collected from a local farm (Ran’s Animal Husbandry) in Youyang County, Chongqing, China (26°54′ N, 108°57′ E). Sixteen clinically healthy castrated male Youzhou black goats (body weight = 15.37 ± 0.58 kg) were randomly sorted into two groups using a totally randomized design approach: (a) the control group (FR, basal diet); (b) the dietary garlic skin group (NR) who were fed a basal diet supplemented with 16% garlic skin on a dry matter basis. The garlic skin originated from the locally cultivated white-skinned garlic in Chongqing and exhibited broad-spectrum antibacterial properties. The animals of the two groups were placed in individual stalls with free-flowing water available. Throughout the 60-day feeding trial period, every animal was fed two times daily, at 07:00 am and 19:00 pm, respectively, with an average daily intake of 2.3–2.5 kg of diet per goat.

### 2.1. Analytical Methods for Basal Diet Composition and Nutritional Profiling

The experimental diet formulation was designed according to the feed standard of meat-producing sheep and goats (NY/T 816-2004). The basal diet includes concentrated feed and forage (garlic skins mixed), and the proportions of each component are shown in Table 1, respectively. The nutritional components of garlic peels can be found in the annex of Supplementary Materials (Supplementary Table S1).

Nutrient levels in feeds are determined according to Chinese national standards (GB) as follows: Dry matter is measured via gravimetric loss at 105 ± 2 °C (GB/T 6435-2014); gross energy uses an adiabatic bomb calorimeter (GB/T 24319-2009); metabolizable energy is calculated from fecal–urine energy losses in animal trials (GB/T 18823-2010); crude protein employs the Kjeldahl method (GB/T 6432-2018), converting nitrogen to ammonium sulfate for titration; neutral/acid detergent fibers are determined by boiling with specific detergents followed by gravimetric analysis (GB/T 20806-2022/GB/T 20805-2006); calcium uses EDTA complexometric titration (GB/T 6436-2002); and total phosphorus is measured via spectrophotometry after acid digestion (GB/T 6437-2018). These methods ensure compliance with national standards, require sample homogenization (e.g., sieving), and allow rapid screening via near-infrared spectroscopy (e.g., DB21/T 2048–2012), with data rounded per GB/T 8170-2008 and validated using reference materials for accuracy.

### 2.2. Slaughter and Meat Quality Indicators

The initial body weight (W0) of the goats was recorded before the start of the feeding trial; after the feeding trial was concluded, the experimental animals were subjected to a 24 h fasting period and a 12 h water deprivation period prior to slaughter. The animals were weighed and we recorded their pre-mortem live weight (W1) and calculated total body weight gain (TWG) and the average daily gain (ADG), using the following formula:

TWG (kg) = W1 − W0(1)

ADG (g/d) = (W1 − W0)/feeding trial days(2)

Subsequently, six goats were randomly selected from each group and their identification numbers were carefully recorded. The slaughter procedures strictly adhered to ethical guidelines and complied with national animal welfare standards for animal slaughter (GB/T 42304-2023), ensuring immediate loss of consciousness through the CO_2_ stunning and a painless termination process. Immediately after slaughter, approximately 250 g of muscle tissue was excised from the mid-section of the longissimus dorsi muscle on the left-hand side of the goat carcass. After removing all visible adipose tissue and connective structures, the collected muscle tissue was carefully divided into two separate parts. One part was kept at 4 °C for 24 h to measure meat quality indicators, such as pH, the color of muscle, cooking loss, etc.; the other part, after rapid freezing in liquid nitrogen, was preserved at −80 °C for metabolomics analysis.

Post-slaughter processing involved the removal of the heads, hooves, fur, and visceral organs, with renal tissue retained for subsequent analysis. The carcass weight (W2) was recorded within 30 min using standardized gravimetric protocols. The dressing percentage was determined using the following formula:

Dressing Percentage (%) = (W2/W1) × 100%(3)

The longissimus dorsi muscle spanning the 12th and 13th intercostal spaces was demarcated using transparent paper, with subsequent quantification of the eye muscle area achieved through the following computation:

Eye muscle area (cm^2^) = eye muscle width × eye muscle height × 0.7 (4)

Backfat thickness (BFT) was measured at the 12th–13th rib level using a vernier caliper (±0.1 mm precision), perpendicular to the long axis of the carcass.

The carcass fat content (GR) value was calculated as the depth of subcutaneous tissue at the midpoint of the 12th–13th rib interface. Metrological assessments were conducted using ISO 9001-certified calipers, achieving 0.1 mm precision through triplicate measurements [14].

The muscle pH quantification was performed with a calibrated handheld portable pH meter (testo 205—pH meter, testo). Samples were collected at 0 h and 24 h after slaughter, and the probe was cleaned with ultrapure water according to the determination method of Ma et al. [15] and pH meter was calibrated before measurement. When measuring, the probe was completely in contact with the muscle sample at 20 °C, the test was repeated three times and the average value was then recorded.

The color of muscle samples was measured using a portable Colorimeter (3nh NR20XE Large aperture Colorimeter, 3nh). Samples were collected at 0 h and 24 h after slaughter. The values of lightness (L*), redness (a*), and yellowness (b*) for the longissimus dorsi muscle were recorded at three distinct locations on the muscle’s surface, after which the mean values were computed [16].

The muscle samples were trimmed into 2 × 2 × 2 cm^3^, weighed (W1), and then each sample was individually suspended using sterile surgical thread within an inflated plastic bag. The muscles were suspended in the center to avoid contact with the bag and kept at 4 °C for 24 h. After using filter paper to remove the surface moisture, the sample was reweighed (W2). The drip loss was determined using the following formula [16]:

Drip loss (%) = [(W1 − W2)/W1] × 100%(5)

Approximately 20 g of muscle samples was taken; after recording the weight of the samples (W3), they were placed in polyethylene bags. Subsequently, the samples were heated in a water-bath pot at 80 °C for 15 min. After heating, the samples were taken out and cooled to room temperature. The samples were then wrapped in filter paper to absorb the surface moisture and weighed and recorded again (W4). The cooking loss was determined using the following formula (NY/T 2793-2015):

Cooking loss (%) = [(W3 − W4)/W3] × 100%(6)

Approximately 20 g of muscle samples was taken. After recording the weight of the samples (W5), they were placed in polyethylene bags. Subsequently, the samples were placed in a freezing apparatus set at −4 °C and left frozen for 24 h. After freezing, the samples were taken out and naturally returned to room temperature. The samples were then wrapped in filter paper to absorb the surface moisture and weighed and recorded again (W6). The freezing loss was determined using the following formula:

Freezing loss (%) = [(W5 − W6)/W5] × 100%(7)

### 2.3. Analysis of the Amino Acid Composition

An appropriate amount of muscle samples was weighed, cut into 1 cm^3^ meat cubes, placed in a watch glass for a steam bath, put into an oven with the juice at 80 °C for drying, and then ground in a grinder. Next, 100 mg of the crushed samples was weighed and put into the hydrolysis tubes, mixed with 5 mL of a 1:1 hydrochloric acid solution, and hydrolyzed in an electrothermal air-blowing oven at 110 ± 1 °C for 22 h. After cooling to room temperature, the hydrolysate was filtered into a 10 mL volumetric flask. The hydrolysis tube was rinsed repeatedly with small volumes of water, and the rinsate was combined into the same flask [17]. The solution was diluted to the mark with water and thoroughly mixed. A 0.05 mL aliquot of the filtrate was transferred to a 15 mL centrifuge tube, dried under a nitrogen stream, and reconstituted to 2 mL with 0.02 mol/L hydrochloric acid. The solution was vortexed, filtered through a 0.22 μm membrane filter, and subjected to the analysis of HPLC (High-Speed Amino Acid Analyzer LA8080 AminoSAAYA, HITACHI, Tokyo, Japan) [18].

### 2.4. Analysis of the Fatty Acid Composition

We weighed 0.5 g of homogenized muscle and placed it into a hydrolysis flask, and added 100 mg pyrogallic acid, boiling stones, and 2 mL of 95% ethanol. After mixing, 10 mL of 6 M hydrochloric acid was added. The mixture was incubated in a 70–80 °C water bath for 40 min, agitated every 10 min, and then cooled to room temperature. The hydrolysate was mixed with 10 mL of 95% ethanol and transferred to a separatory funnel. The flask was rinsed thrice with 50 mL of a 1:1 diethyl ether–petroleum ether mixture and the rinsate was added to the funnel. After 5 min of shaking and 10 min of phase separation, the ether layer was collected in a pre-weighed 250 mL flask; this extraction was repeated twice. The pooled ether was evaporated at 60 °C under reduced pressure, and the residual lipids were dried at 100 ± 5 °C for 2 h. The extracted lipids were saponified with 2 mL of 2% NaOH in methanol at 85 °C for 30 min. Then, 3 mL of 14% boron trifluoride-methanol was added to synthesize fatty acid methyl esters (FAMEs), followed by another 30 min incubation at 85 °C. After cooling, 3 mL of n-hexane was added, vortexed for 2 min, and left for 1 h of phase separation. The FAME-containing hexane layer was filtered through a 0.45 μm PTFE membrane before GC analysis. GC was performed on an Agilent 7890B with an HP-88 column (100 m × 0.25 mm × 0.20 μm) and FID. The oven’s temperature started at 100 °C (15 min hold), increased to 190 °C at 20 °C/min (6 min hold) and then to 220 °C at 1 °C/min (7 min hold). Injector and detector temperatures were 260 °C and 250 °C. High-purity nitrogen was the carrier gas at 1.0 mL/min with a 20:1 split ratio. FAMEs were identified by comparing with Supelco 37 Component FAME Mix standards [19,20].

### 2.5. UPLC-MS/MS Metabolomic Analysis

Metabolite extraction products from black goat muscle samples were subjected to UPLC-MS/MS analysis (Waters platform: ACQUITY I-Class Plus + Thermo Scientific Q Exactive Plus HRMS, Waters Technologies (Shanghai) Limited, Shanghai, China) under negative ion mode. Following a standardized pretreatment protocol, 30 mg aliquots of muscle tissue were precisely weighed (±0.01 mg) into 1.5 mL tubes containing 400 μL ice-cold methanol–water (4:1 *v*/*v*, containing 4 μg/mL internal standards) and stainless steel grinding beads. After pre-cooling at −40 °C for 2 min, samples were homogenized (45 Hz, 2 min) followed by ultrasonic-assisted extraction in an ice-water bath (10 min) and overnight metabolite solubilization at −40 °C. The extracts were centrifuged (12,000× *g*, 20 min, 4 °C) with 150 μL supernatant transferred to LC-MS vials for analysis. Quality control (QC) samples were prepared by pooling 10 μL aliquots from each extract for system conditioning and data quality monitoring. Chromatographic resolution was performed on an ACQUITY UPLC HSS T3 analytical column (2.1 mm i.d. × 100 mm length, 1.8 μm particle size) (Waters Technologies (Shanghai) Limited, Shanghai, China) employing a binary mobile phase system of 0.1% (*v*/*v*) aqueous formic acid and acetonitrile. The elution gradient was maintained at 0.35 mL/min with column thermostatting to 45 °C. Mass spectrometric detection employed a Q Exactive Plus Orbitrap (Thermo Fisher Scientific (China) Co., Ltd., Shanghai, China) operated in polarity switching mode, enabling high-resolution separation and accurate mass measurements (<3 ppm) for comprehensive metabolite identification [21,22].

### 2.6. Statistical Analysis

The experimental data were collated and analyzed, individual black goats were considered experimental units, and the differences in various indicators between the two groups of black goats were compared. Microsoft Excel 2016 software was used to preliminarily organize the experimental data, followed by statistical analysis using independent samples *t*-tests implemented in SPSS 26.0 (SPSS Inc., Chicago, IL, USA). Results were presented as mean ± standard deviation. Differences were considered significant when *p* < 0.05, and *p* > 0.05 indicated no significant difference.

Multivariate statistical analysis was performed to identify differential metabolites and perturbed pathways. Initially, unsupervised principal component analysis (PCA) was employed within this framework. PCA allowed us to observe the overall distribution among samples as well as the stability of the entire analysis procedure. Subsequently, supervised partial least squares analysis (PLS-DA) and orthogonal partial least squares analysis (OPLS-DA) were utilized. These techniques were applied to discern the overall disparities in metabolic profiles between groups and to uncover the differential metabolites present between the FR and the NR group [22,23].

## 3. Results

### 3.1. Comparison of Slaughter Performance Indicators of Two Groups of Goats

Based on the data provided in Table 2, no significant differences were observed in live weight, TWG, ADG, carcass weight, and dressing percentage between the NR and the FR group (*p* > 0.05). However, the NR group showed notably lower BFT than those in the FR group (*p* < 0.05), and the eye muscle area of the NR group showed significantly higher than those of the FR group (*p* < 0.05).

### 3.2. Comparison of Meat Quality Indicators of Two Groups of Goats

As shown in Table 3, after slaughter, the pH value of the NR group at 0 h was significantly higher than that of the FR group (*p* < 0.05); After 24 h, the pH value of both groups decreased, but there was no significant difference (*p* > 0.05). However, there was no significant difference between the brightness (L*), redness (a*), and yellowness (b*) parameters of the NR group and the FR group at 0 h (*p* > 0.05). After 24 h, the above values of the two groups increased, but only L* and a* of the NR group were significantly higher than that of the FR group (*p* < 0.05), and b* was not significantly different (*p* > 0.05). In addition, the parameters of drip loss, cooking loss, and freezing loss in the NR group were slightly lower than those in the FR group, but the difference was only statistically significant in cooking loss (*p* < 0.05).

### 3.3. Comparison of Amino Acid and Fatty Acid Indicators of Two Groups of Goats

Comparative analysis of amino acid (AA) and fatty acid (FA) profiles between the two groups revealed distinct compositional variations (Table 4 and Table 5). Compared with the NR group, the FR group exhibited marked reductions (*p* < 0.05) in specific AAs, including asparagine, serine, leucine, glutamic acid, and lysine. Notably, arginine levels in FR were lower than in NR (*p* < 0.01). For FAs (Table 5), lipidomic profiling demonstrated NR’s nutritional superiority, with higher total n-3 PUFAs (*p* < 0.01), driven by elevated α-linolenic acid (ALA, 18:3n3), eicosapentaenoic acid (EPA, 20:5n3), and docosahexaenoic acid (DHA, 22:6n3). In contrast, FR displayed higher concentrations of γ-linolenic acid (GLA, 18:3n6) and 2-decyltetradecanoic acid (*p* < 0.05). The NR group’s FA profile showed increased health-beneficial components, i.e., higher cis-11-Eicosenoic acid and saturated fatty acids (SFAs), including butyric acid (*p* < 0.05), caprylic acid (*p* < 0.05), eicosanoic acid (*p* < 0.01) and tricosanoic acid (*p* < 0.01), alongside reduced n-6/n-3 ratios. These compositional shifts, particularly the n-3 PUFA enrichment, confirm garlic skin’s efficacy in enhancing goats’ nutritional value through targeted lipid modulation and redox balance optimization and highlight the potential of dietary interventions to modulate meat composition for improved functional properties.

### 3.4. Untargeted Metabolomics Profiling of Meat

#### 3.4.1. Overview of Metabolomics Analysis

**Figure 1:**
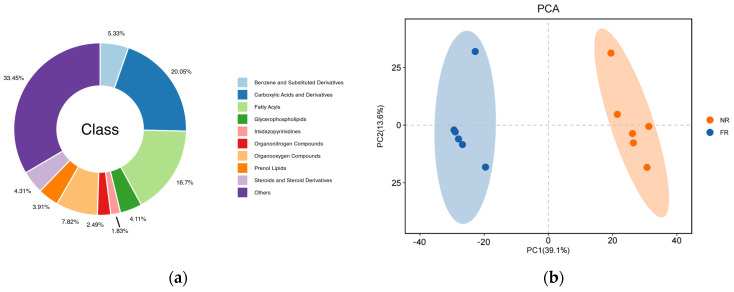

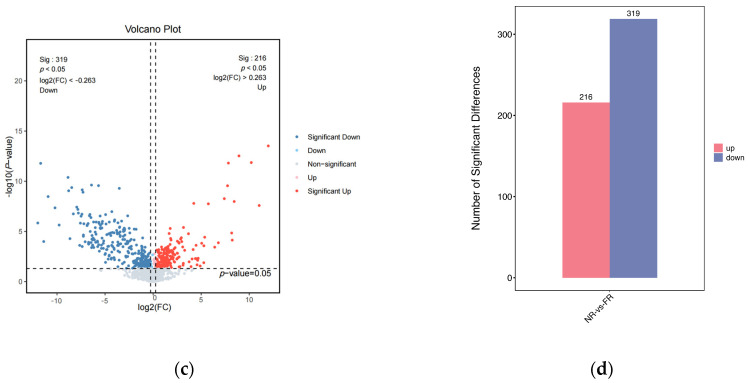
(**a**) The classification of the nontargeted metabolomics of the two groups; (**b**) the score plots of PCA plots; (**c**) the volcano plot of the significantly differentially abundant metabolites. Red dots represent metabolites that were up-regulated in the experimental group, blue dots represent metabolites that were down-regulated, and gray dots represent metabolites that were not significant, with the horizontal coordinate being the log2 (FC) value for the comparison of the two groups, and the vertical coordinate being the −log10 (*p*-value) value. (**d**) The histogram of differential metabolite counts.

To elucidate the effect of the dietary addition of garlic skin on the molecular changes in goat meat, the metabolite compositions of two goat samples (n = 6) were analyzed using LC-MS/MS. In total, as shown in Figure 1a, 1970 metabolites were detected in the goat meat samples, and the metabolites could be classified into 10 groups: carboxylic acids and derivatives (20.05%); fatty acyls (16.7%); organooxygen compounds (7.82%); benzene and substituted derivatives (5.33%); steroids and steroid derivatives (4.31%); glycerophospholipids (4.11%); prenol lipids (3.91%); organonitrogen compounds (2.49%); lmidazopyrimidines (1.83%); others (33.45%).

As shown in Figure 1b, the 7-fold cross-validated PCA score plot demonstrated tight clustering of QC samples, confirming experimental reproducibility and result reliability. Distinct separation between FR and NR groups revealed statistically significant intergroup metabolite differences. OPLS-DA analysis revealed a distinct metabolite separation pattern between the NR and FR groups. The model validation parameters R^2^Y and Q^2^Y exhibited linear trajectories with pronounced slopes, and all Q^2^ validation points had values lower than their original counterparts (results shown in Supplementary Figure S1a,b). These findings indicate that the separation of metabolites among groups was statistically significant.

As shown in Figure 1c, using volcano plots to visualize *p*-values and fold change (FC) values is beneficial for screening differential metabolites. The red and blue dots represent the significantly different metabolites after screening. Significantly differentially abundant metabolites were further screened based on variable importance in projection (VIP) > 1, *p*-value < 0.05, and FC ≥ 1.2 or FC ≤ 1/1.2. In the comparison of the FR group vs. the NR group (Figure 1d), a total of 535 metabolites exhibited significant differential regulation. Among these, 319 metabolites demonstrated upregulation, while 216 metabolites showed downregulation.

#### 3.4.2. KEGG Enrichment Analysis

**Figure 2:**
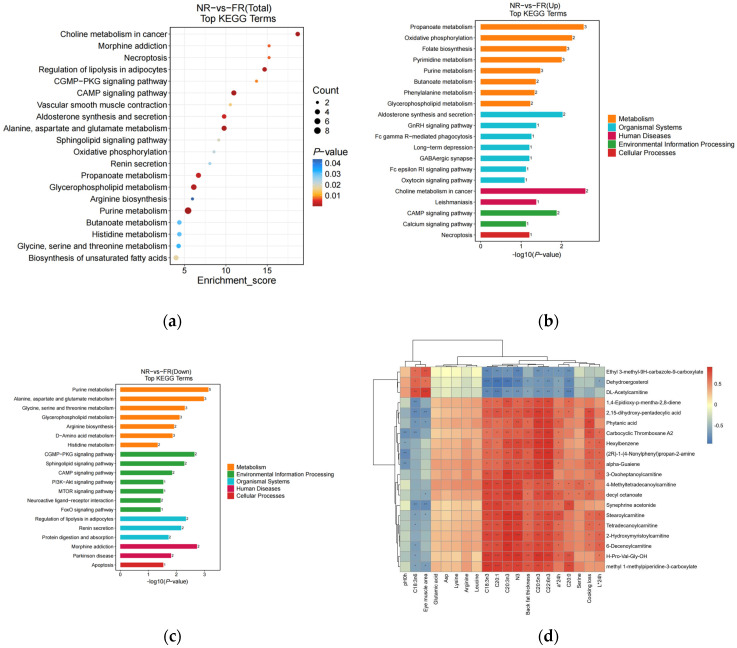
(**a**) A bubble diagram was constructed to display the top 20 enriched KEGG pathways resulting from the comparison between the two groups. Each bubble in the diagram corresponds to a metabolic pathway. The size of the bubbles is proportional to the impact factor, and the bubble color depth is proportional to the degree of enrichment, such that a larger and darker-colored bubble indicates a greater impact factor and more significant degree of enrichment. (**b**,**c**) The histograms of the top 20 enriched KEGG Level 3 distribution (up and down) of differential metabolites between the two groups. The horizontal coordinate is the −log10 *p*-value for each pathway, the vertical coordinate is the name of the different pathways, the number on the bar is the number of differential metabolites annotated to that pathway, and the different colors of the bar represent different KEGG Level 1 information. (**d**) A correlation heat map cluster between meat quality parameters and differential muscle metabolites. Each column represents a different species, and each row corresponds to a specific metabolite. Orange-red indicates positive correlations, while blue represents negative correlations. Darker colors signify stronger correlations, whereas lighter colors denote correlations closer to zero. *** *p* < 0.001, ** *p* < 0.01, and * *p* < 0.05.

To gain a deeper understanding of the pathways related to the differentially abundant metabolites, differential metabolites screened under both positive and negative ion modes were integrated first. Figure 2a and Supplementary Table S3 visualize the 20 most enriched metabolic pathways derived from discriminant metabolite analysis. Among these, most of these pathways were associated with amino acid metabolism, lipid metabolism, the endocrine system, vascular smooth muscle contraction, signaling pathways, carbohydrate metabolism, cell growth and death, and nucleotide metabolism. These were the significant pathways influenced by the meat quality of the goat (Figure 2a). Comparative pathway flux analysis under divergent feeding regimens was conducted through differential abundance scoring (Figure 2b,c), a computational approach quantifying the average and overall alterations of all metabolites by integrating magnitude and directional consistency of metabolite-level changes within each pathway.

The major up-regulated metabolic pathways thus identified were propanoate metabolism, oxidative phosphorylation, folate biosynthesis, pyrimidine metabolism, aldosterone synthesis and secretion, cAMP signaling pathway, purine metabolism, butanoate metabolism, phenylalanine metabolism, and glycerophospholipid metabolism. In the same vein, the down-regulated metabolic pathways encompassed purine metabolism, alanine, aspartate and glutamate metabolism, glycine, serine, and threonine metabolism, glycerophospholipid metabolism, cGMP-PKG signaling pathway, sphingolipid signaling pathway, regulation of lipolysis in adipocytes, renin secretion, morphine addiction, and arginine biosynthesis. 

#### 3.4.3. Analysis of the Correlation Between the Metabolites Related to the Differences in Meat Quality and Muscle

To investigate the relationship between muscle metabolism and meat quality in black goats under different feeding regimens, we performed a correlation analysis between meat quality parameters and non-targeted metabolomics data. This approach allowed us to quantify the associations among significantly differential metabolites and elucidate their interactions during biological state transitions. The top 50 differentially abundant metabolites with the smallest *p*-values were selected (Supplementary Table S4) and their correlations with meat quality indicators, amino acids, and fatty acids showing significant differences in prior analyses were evaluated. The results revealed strong correlations between these metabolites and phenotypic traits, with the top 20 correlations visualized in Figure 2d. In particular, meat color indices L* and a* were positively correlated with Carbocyclic Thromboxane A2, Hexylbenzene, (2R)-1-(4-Nonylphenyl)propan-2-amine, alpha-Guaiene, Stearoylcarnitine, Tetradecanoylcarnitine, 2-Hydroxymyristoylcarnitine, 6-Decenoylcarnitine and H-Pro-Val-Gly-OH, but negatively correlated with Dehydroergosterol. In addition, a* was also positively correlated with 2,15-dihydroxy-pentadecylic acid, phytanic acid, 4-Methyltetradecanoylcarnitine, decyl octanoate and Synephrine acetonide, but negatively correlated with Ethyl 3-methyl-9H-carbazole-9-carboxylate, Dehydroergosterol and DL-Acetylcarnitine. L* was also positively correlated with 3-Oxoheptanoylcarnitine.

There was also a positive correlation between Eicosanoic acid (C20:0) and methyl 1-methylpiperidine-3-carboxylate, H-Pro-Val-Gly-OH, Synephrine acetonide, 4-Methyltetradecanoylcarnitine and decyl octanoate. Eicosanoic acid was negatively correlated with Ethyl 3-methyl-9H-carbazole-9-carboxylate, Dehydroergosterol and DL-Acetylcarnitine. Serine had only a positive correlation with 4-Methyltetradecanoylcarnitine and decyl octanoate. There was a positive correlation between cooking loss and 2,15-dihydroxy-pentadecylic acid, phytanic acid, Carbocyclic Thromboxane A2, Hexylbenzene, (2R)-1-(4-Nonylphenyl)propan-2-amine, alpha-Guaiene, 4-Methyltetradecanoylcarnitine, decyl octanoate, Stearoylcarnitine, Tetradecanoylcarnitine, 2-Hydroxymyristoylcarnitine and 6-Decenoylcarnitine.

Omega-3 polyunsaturated fatty acids (n-3 PUFA), including α-linolenic acid, eicosapentaenoic acid (EPA), and docosahexaenoic acid (DHA), along with C20:1 and backfat thickness, were positively correlated with 1,4-epidioxy-p-mentha-2,8-diene, 2,15-dihydroxy-pentadecylic acid, phytanic acid, carbocyclic thromboxane A2, hexylbenzene, (2R)-1-(4-nonylphenyl)propan-2-amine, alpha-guaiene, 3-oxoheptanoylcarnitine, 4-methyltetradecanoylcarnitine, decyl octanoate, synephrine acetonide, stearoylcarnitine, tetradecanoylcarnitine, 2-hydroxymyristoylcarnitine, 6-decenoylcarnitine, H-Pro-Val-Gly-OH, and methyl 1-methylpiperidine-3-carboxylate. Conversely, these were negatively correlated with dehydroergosterol and DL-acetylcarnitine.

The eye muscle area was positively correlated with Ethyl 3-methyl-9H-carbazole-9-carboxylate, dehydroergosterol, and DL-acetylcarnitine, and negatively correlated with 2,15-dihydroxy-pentadecylic acid, phytanic acid, decyl octanoate, synephrine acetonide, stearoylcarnitine, and methyl 1-methylpiperidine-3-carboxylate. Gamma-linolenic acid (C18:3n6) was positively correlated with Ethyl 3-methyl-9H-carbazole-9-carboxylate, dehydroergosterol, and DL-acetylcarnitine, and negatively correlated with 1,4-epidioxy-p-mentha-2,8-diene, 2,15-dihydroxy-pentadecylic acid, phytanic acid, carbocyclic thromboxane A2, synephrine acetonide, stearoylcarnitine, tetradecanoylcarnitine, 6-decenoylcarnitine, H-Pro-Val-Gly-OH, and methyl 1-methylpiperidine-3-carboxylate. Additionally, pH_0h_ was negatively correlated only with carbocyclic thromboxane A2, hexylbenzene, (2R)-1-(4-nonylphenyl)propan-2-amine, and alpha-guaiene.

## 4. Discussion

The quality of the meat is important for consumer acceptance of that meat product. In this study, no significant differences were detected in live weight, carcass weight, and slaughter rate when comparing the control group (FR) with the dietary garlic skin group (NR). These findings align with those of the research regarding the impact of dietary supplementation of garlic stem and husk on lamb performance and carcass characteristics [24]. Still, in actual feeding, we found that the addition of garlic skin tended to increase the feed intake of the goats, which is consistent with the results of the previous study in which the addition of phyto-organic compounds (plant-based raw materials or extracts) can improve feeding efficiency, and Savairam et al. found that garlic and its by-products emit a unique aroma that stimulates appetite, enhancing feed intake, and promoting digestion by increasing gastric juice secretion and gastrointestinal motility [25,26], which may be due to the addition of garlic skin improving the odor or taste of the feed. The significant decrease in backfat thickness (BFT) and a significant increase in eye muscle area in the NR group suggests that garlic skin may regulate fat metabolism or inhibit adipogenesis and promote myofibril development through its bioactive constituents (e.g., allicin and organosulfur derivatives) to improve the carcass composition. It is now well established that allicin can regulate lipid metabolism by inhibiting adipocyte differentiation through the AMPK pathway [27], and organosulfur compounds (e.g., diallyl disulfide) in garlic have also been shown to inhibit adipocyte differentiation and activate the AMPK pathway, which reduces fat deposition and promotes muscle growth [28]. In addition, it has been demonstrated that allicin reduces fat synthesis by inhibiting acetyl-CoA synthetase activity, thereby decreasing backfat thickness [29]. The increase in eye muscle area may be related to the promotion of muscle protein synthesis by polyphenols in garlic skin, the mechanism of which may associated with the activation of the mTOR signaling pathway [30].

In terms of meat quality parameters, a higher pH value indicates a faster oxidative metabolic rate and elevated heme content. In this study, the pH value of the NR group at 0 h post-slaughter was significantly higher than that of the FR group. This phenomenon may be attributed to polyphenols in garlic skin enhancing antioxidant capacity by increasing the activity of muscle superoxide dismutase (SOD), while inhibiting key glycolytic enzymes (e.g., phosphofructokinase), thereby delaying glycogenolysis and maintaining postmortem muscle glycogen reserves [31,32]. These findings align with previous reports that garlic polyphenols suppress lactic acid accumulation through antioxidative mechanisms [33]. After 24 h, the pH values of both groups declined, which is explained by the shift from aerobic to anaerobic respiration in post-slaughter muscles, where glycogen is catabolized into lactic acid, leading to a significant pH reduction [34]. However, the absence of intergroup pH differences at 24 h suggests that garlic skin primarily influences meat pH during the early postmortem period. The higher initial pH in the NR group likely contributed to preserving the structural integrity of muscle proteins, thereby improving meat tenderness. Although the pH difference between groups diminished after 24 h, the early pH advantage in the NR group may have delayed protein denaturation.

Meat color is the primary sensory indicator for consumers to assess freshness. A higher L* value indicates greater lightness (whitish color), while a higher a* value corresponds to increased redness (bright red), and a lower a* value suggests a greenish hue. The b* value reflects yellowness, with higher values indicating more yellow and lower values leaning toward blue. Although no significant differences in meat color parameters (L*, a*, b*) were observed between the two groups at 0 h, the NR group exhibited higher L* and a* values compared to the FR group at 24 h. This aligns with findings by Liao et al., who reported that garlic byproducts significantly enhanced redness (a*) in chicken meat, suggesting universal applicability of garlic-derived supplements in improving livestock meat quality [35]. The superior color retention in the NR group may be attributed to the antioxidant properties of garlic polyphenols. Studies indicate that polyphenols in garlic skin, such as quercetin and thiosulfinates, effectively scavenge free radicals and inhibit the oxidation of myoglobin to metmyoglobin, thereby maintaining color stability [36]. Furthermore, garlic saponins may affect phosphorylation levels and protect against oxidative stress-induced cellular damage, a conclusion consistent with the metabolomic findings of activated oxidative phosphorylation pathways in this study, further implying the effects of garlic skin on meat [37]. The enhanced preservation of lightness and redness reflects garlic’s antioxidant capacity, mediated through phytanic acid (PA), which participates in adipocyte differentiation, and dehydroergosterol, which influences lipid metabolism. These compounds mitigate free radical damage and delay myoglobin oxidation, stabilizing meat color [38,39]. Correlation analysis further revealed positive associations between L*/a* values and metabolites such as tetradecanoylcarnitine and stearoylcarnitine, suggesting that these acylcarnitines may regulate mitochondrial β-oxidation, TCA cycle efficiency, and oxidative phosphorylation to maintain energy homeostasis, indirectly benefiting color stability. This validates garlic skin’s role in enhancing oxidative stability [40].

Although no statistical differences were observed in drip loss or freezing loss between groups, notably, cooking loss in the NR group was significantly reduced compared to the FR group (*p* < 0.05), consistent with studies demonstrating that natural plant antioxidants in diets improve water retention and cooking loss in rabbit meat [41]. This may be linked to allicin’s ability to inhibit mitochondrial dysfunction and apoptosis via signaling pathways, implying that garlic skin reduces fluid loss by enhancing mitochondrial function [42]. Additionally, metabolomic analysis revealed upregulation of glycerophospholipid metabolism in the NR group. Products of this pathway, such as phosphatidylcholine, may strengthen myocyte membrane integrity, reducing postmortem water loss [43].

The content and variety of amino acids directly influence the flavor and texture of meat. Amino acid analysis revealed significantly lower levels of aspartic acid, serine, glutamic acid, leucine, and lysine in the FR group compared to the NR group. Notably, arginine (Arg) content in the FR group was markedly lower than in the NR group. Arginine not only serves as a critical precursor for collagen synthesis but also regulates vasodilation via the nitric oxide (NO) pathway, reducing oxidative damage and indirectly improving meat tenderness [44]. Lysine, a common amino acid in meat, enhances umami flavor and reduces cooking loss by improving water-holding capacity, consistent with the findings above. The higher lysine content in the NR group indicates superior meat quality [45]. Leucine, an essential branched-chain amino acid that must be supplied through the diet, modulates the mammalian target of the rapamycin (mTOR) signaling pathway in muscle cells, promoting protein synthesis. Additionally, it improves body fat composition in pigs by altering gut microbiota, thereby enhancing meat quality [46]. Furthermore, leucine modifies serum metabolism to promote muscle growth and reduce fat deposition, which is why it is typically one of the most abundant amino acids in high-quality protein foods [47].

Dietary n-3 polyunsaturated fatty acids (n-3 PUFAs), including α-linolenic acid (ALA), eicosapentaenoic acid (EPA), and docosahexaenoic acid (DHA), are essential fatty acids for humans. They play critical roles in hypolipidemic and hypotensive effects, cholesterol modulation, prevention of cardiovascular diseases, and promotion of brain and nervous system development. Consequently, consumers prioritize food sources rich in n-3 PUFAs [48,49,50]. However, mammals cannot synthesize n-3 polyunsaturated fatty acids (PUFAs) endogenously and can only ingest and utilize them from food. Current studies have explored enhancing animal growth performance and n-3 PUFA content while reducing production costs by either directly adding n-3 PUFAs to feed or indirectly incorporating fish oil or flaxseeds. Garlic skin, meanwhile, offers the potential to serve as a non-traditional enhancer of n-3 PUFAs [51,52]. In this study, the NR group exhibited a significant increase in n-3 PUFA content. The elevated ALA levels suggest that garlic skin may enhance the conversion efficiency of linolenic acid by rumen microbiota, thereby promoting the synthesis of long-chain n-3 PUFAs (e.g., EPA and DHA). This phenomenon could be attributed to both the direct supplementation of PUFAs from garlic skin and its inhibitory effects on fatty acid desaturase activity [53,54,55]. The enrichment of EPA and DHA directly enhances the functional nutritional value of mutton, aligning with consumer demands for anti-inflammatory and cardiovascular health benefits. Short-chain fatty acids (SCFAs) have multiple biological functions, such as participating in the metabolic regulation of the body, promoting the development of the gastrointestinal tract, and regulating the microbial community of the gastrointestinal tract. Current studies have found that SCFAs can regulate lipid metabolism by activating PPARγtranscription factors through the rumen–muscle axis, thereby influencing sheep meat quality [56]. This is consistent with the result in this study that the C4:0 content in the NR group was significantly higher than that in the FR group. Notably, the significant increase in C20:3n3 also may improve lipid metabolism by modulating the PPARγ pathway [57].

Furthermore, the reduced n-6/n-3 ratio in the NR group aligns with the WHO-recommended dietary fatty acid profile, contributing to lowered blood lipid levels, cardiovascular disease prevention, and improved quality of life [58,59]. Of particular interest is the elevated content of long-chain SFAs such as C20:0 (arachidic acid) in the NR group, likely due to garlic skin’s inhibition of acetyl-CoA carboxylase (ACC) activity, which is the rate-limiting enzyme in fatty acid synthesis [29]. Concurrently, the increase in short-chain SFAs (e.g., butyric acid and caprylic acid) may reflect shifts in rumen microbial fermentation patterns [60]. These findings collectively indicate a transition toward a healthier lipid profile. Metabolomic correlation analysis further supports these conclusions. For instance, the positive correlation between carnitine derivatives (e.g., tetradecanoylcarnitine) and cooking loss suggests that enhanced mitochondrial β-oxidation may accelerate energy metabolism, indirectly affecting water-holding capacity [40].

Metabolomic analysis identified 1970 differentially abundant metabolites, predominantly enriched in pathways such as propanoate metabolism, oxidative phosphorylation, and amino acid metabolism. Dietary supplementation with garlic skin in the NR group significantly upregulated propanoate metabolism, oxidative phosphorylation, glycerophospholipid metabolism, and folate biosynthesis. These pathways are closely associated with energy production and membrane lipid remodeling, a process potentially driven by garlic-derived sulfur compounds through modulation of enzyme activities. The upregulation of propanoate metabolism may enhance ATP generation in the tricarboxylic acid (TCA) cycle, thereby optimizing muscle energy status [61]. In propanoate metabolism, propionate likely acts as a gluconeogenic precursor to promote hepatic glycogen synthesis, delaying postmortem pH decline [62]. Activation of oxidative phosphorylation indicates enhanced mitochondrial function, consistent with previous findings that garlic polysaccharides improve the activity of mitochondrial respiratory chain complexes [37]. Enrichment of folate biosynthesis facilitates one-carbon metabolism, which neutralizes free radicals via glutathione (GSH) synthesis, protects lipids and myoglobin from oxidative damage, and supports methylation reactions critical for muscle development (e.g., DNA modification, carnitine synthesis) [63]. Additionally, propionyl-CoA, a key intermediate in propanoate metabolism, participates in odd-chain fatty acid synthesis (e.g., C15:0, C17:0), potentially influencing membrane lipid composition. Conversely, the downregulation of purine metabolism and alanine/aspartate/glutamate pathways may reflect reduced proteolysis or oxidative stress, aligning with garlic’s antioxidative properties. The bidirectional regulation of glycerophospholipid metabolism (partial up- and downregulation) suggests a homeostatic role of garlic skin in membrane lipid remodeling: upregulation may increase phosphatidylethanolamine (PE) levels, enhancing membrane fluidity due to high n-3 PUFA content [64], while downregulation of sphingolipid metabolism could reduce pro-apoptotic signals (e.g., ceramides), preserving myocyte integrity [65].

Correlation analysis revealed positive associations between meat color parameters (L, a) and metabolites such as carbocyclic thromboxane A2 and phytanic acid, indicating that these compounds may regulate inflammation by inhibiting lipoxygenase (LOX) activity, thereby delaying meat discoloration [66]. The negative correlation between n-3 PUFAs and dehydroergosterol implies that steroidal metabolites may competitively bind desaturases, suppressing n-3 PUFA synthesis [67].

While this study confirms the beneficial effects of garlic skin on meat quality, the precise mechanisms linking metabolomic changes to quality improvements in black goat meat remain incompletely defined. Future research should focus on interactions between garlic skin bioactive components and gut microbiota, as well as establish dose–response relationships for specific constituents to further elucidate the regulatory mechanisms of lipid and amino acid metabolism.

## 5. Conclusions

This metabolomics study revealed that dietary garlic skin supplementation enhanced black goat meat quality by modulating carcass composition and metabolic pathways. Garlic skin reduced backfat thickness, expanded eye muscle area, and improved post-slaughter meat characteristics through delayed pH decline, enhanced color stability (elevated L* and a* values), and reduced cooking loss. Nutritional profiling demonstrated increased arginine, n-3 PUFAs (ALA, EPA and DHA), and short-chain fatty acid production via rumen microbial modulation. The increase in arginine and short-chain fatty acids improves the tenderness of meat by reducing oxidative damage and regulating lipid metabolism, respectively, thereby affecting the quality of goat meat. Metabolomic analysis identified 1970 metabolites, with differential regulation in lipid-related compounds (fatty acyls, glycerophospholipids) and bioactive derivatives. Key enriched pathways included amino acid metabolism, lipid metabolism, and cGMP-PKG signaling and these pathways may regulate meat quality through multiple mechanisms, including enhanced proteolytic regulation via amino acid metabolism, which influences lipid synthesis and metabolism. The cGMP-PKG signaling pathway may reduce the stiffness of corpses after death and maintain color stability. The synergistic effects of these pathways suggesting garlic skin optimizes meat quality through dual regulation of antioxidant activity and energy-lipid homeostasis. While validating garlic skin’s potential as a natural feed additive for functional goat production, further research is needed to elucidate dose–response relationships and gut microbiota interactions. These findings provide a scientific framework for developing phytogenic supplements in sustainable goat farming. Future research could leverage these findings to develop natural feed additives and promote the production of high-quality functional goat meat.

## Figures and Tables

**Table 1:** Diet formulation and nutritional composition.

Items	FR	NR
Corn straw (%)	25	8
Peanut straw (%)	32	32
Garlic skin (%)	-	16
Soybean meal (%)	15	15
Corn (%)	24	24
Sodium chloride (%)	0.5	0.5
Limestone (%)	0.4	1
Dicalcium phosphate (%)	0.7	0.7
Urea phosphate (%)	0.4	0.8
Premix ^1^	2	2
	100	100
Nutrient levels ^2^		
Dry matter (fresh)	33.25	33.31
Organic matter (%)	88.17	87.91
Ash (%)	8.65	8.46
Gross energy (MJ/kg)	15.12	15.20
Metabolizable energy (MJ/kg)	9.16	9.15
Total digestible nutrients (%)	49.78	49.73
Crude protein (%)	14.26	14.3
Neutral detergent fiber (%)	42.62	42.69
Acid detergent fiber (%)	21.20	21.22
Ether extract (%)	3.11	2.98
Non-fiber carbohydrates (%)	31.36	31.57
Calcium (%)	0.94	0.95
Total phosphorus (%)	0.55	0.56

^1^ The premix provides the following per kilogram of feed: Vitamin A (VA) 60,000 IU, vitamin D3 (VD) 39,000 IU, vitamin E (VE) 75 IU, niacin 150 mg, pantothenic acid 45 mg, biotin 3.0 mg, copper (Cu) 10 mg, zinc (Zn) 50 mg, iron (Fe) 70 mg, selenium (Se) 0.30 mg, iodine (I) 5.25 mg, manganese (Mn) 50 mg; cobalt (Co) 2.25 mg. ^2^ ME was a calculated value while the others were measured values.

**Table 2:** The differences in slaughter performance between FR and NR.

	FR	NR	*t*	*p*
Initial weight (kg)	15.57 ± 0.67	15.17 ± 0.43	−1.242	0.242
Fasted live weight (kg)	22.15 ± 3.75	22.56 ± 1.41	0.250	0.808
TWG (kg)	6.57 ± 3.28	7.39 ± 1.30	0.566	0.584
ADG (g/d)	109.56 ± 54.59	123.14 ± 21.69	0.566	0.584
Carcass weight (kg)	10.04 ± 2.11	9.66 ± 1.80	−0.339	0.742
Dressing percentage (%)	45.22 ± 3.85	42.58 ± 5.75	−0.935	0.372
Eye muscle area (cm^2^)	8.02 ± 1.15 *	9.45 ± 0.71	2.593	0.027
GR (mm)	2.84 ± 0.34	3.07 ± 0.28	1.313	0.219
Backfat thickness (mm)	3.14 ± 0.70 *	2.18 ± 0.54	−2.641	0.025

* represents a significant difference (*p* < 0.05); FR: the control group; NR: the dietary garlic skin group; *t*: t-statistic; ADG: average daily gain; TWG: total body weight gain; GR: carcass fat content.

**Table 3:** The differences in meat quality between FR and NR.

	FR	NR	*t*	*p*
pH_0h_	5.56 ± 0.24 *	5.93 ± 0.29	−2.38	0.039
pH_24h_	4.83 ± 0.17	4.85 ± 0.12	−0.14	0.895
L*_0h_	39.49 ± 2.62	41.11 ± 1.45	1.32	0.224
a*_0h_	14.50 ± 0.52	14.57 ± 0.86	0.19	0.855
b*_0h_	8.82 ± 1.30	8.20 ± 0.74	−1.00	0.342
L*_24h_	44.60 ± 3.51 *	48.94 ± 3.10	2.27	0.046
a*_24h_	16.16 ± 1.27 *	18.77 ± 1.97	2.73	0.021
b*_24h_	10.25 ± 0.84	11.37 ± 1.97	1.28	0.243
Drip loss (%)	4.19 ± 2.00	4.12 ± 1.50	−0.58	0.956
Cooking loss (%)	38.26 ± 3.67 **	30.39 ± 4.73	−3.22	0.009
Freezing loss (%)	6.50 ± 2.53	4.41 ± 3.59	−1.17	0.269

* represents a significant difference (*p* < 0.05); ** represents a very significant difference (*p* < 0.01); FR: the control group; NR: the dietary garlic skin group; *t*: t-statistic.

**Table 4:** The differences in amino acid between FR and NR.

AA (g/100 g)	FR	NR	*t*	*p*
Asp	1.78 ± 0.07 *	1.87 ± 0.03	2.59	0.032
Ser	0.73 ± 0.03 *	0.78 ± 0.02	3.311	0.011
Glu	2.94 ± 0.13 *	3.13 ± 0.04	3.029	0.016
Gly	0.90 ± 0.04	0.97 ± 0.08	1.827	0.105
Ala	1.11 ± 0.05	1.14 ± 0.07	0.849	0.416
Val	1.05 ± 0.05	1.04 ± 0.06	−0.272	0.791
Met	0.23 ± 0.08	0.27 ± 0.05	1.159	0.273
IIe	0.93 ± 0.04	0.96 ± 0.01	1.608	0.146
Leu	1.57 ± 0.06 *	1.68 ± 0.04	3.128	0.02
Lys	1.75 ± 0.07 *	1.85 ± 0.04	2.665	0.029
His	0.61 ± 0.08	0.67 ± 0.01	1.655	0.172
Arg	1.24 ± 0.05 **	1.35 ± 0.02	4.663	0.003
EAAs	1.04 ± 0.05	1.06 ± 0.06	0.549	0.595
TAAs	1.08 ± 0.05	1.11 ± 0.06	0.922	0.378

* represents a significant difference (*p* < 0.05); ** represents a very significant difference (*p* < 0.01); FR: the control group; NR: the dietary garlic skin group; *t*: t-statistic; 1 EAA = (leucine + methionine + valine + isoleucine + threonine + phenylalanine + lysine). EAAs: essential amino acids; 2 TAAs = (aspartic acid + threonine + serine + glutamic acid + glycine + alanine + cystine + valine + methionine + isoleucine + leucine + tyrosine + phenylalanine + lysine + histidine + arginine + proline). TAAs: total amino acids.

**Table 5:** The differences in fatty acid between FR and NR.

FA (mg/kg)	FR	NR	*t*	*p*
C4:0	0.60 ± 0.14 *	0.86 ± 0.22	2.455	0.034
C6:0	0.15 ± 0.04	0.19 ± 0.06	1.343	0.209
C8:0	0.52 ± 0.13 *	0.74 ± 0.19	2.256	0.048
C10:0	5.11 ± 2.09	4.49 ± 0.26	−0.457	0.657
C12:0	2.90 ± 1.39	4.26 ± 4.36	0.728	0.483
C18:1n9c	5869.83 ± 1933.81	3972.88 ± 1428.89	−1.932	0.082
C18:2n6c	648.65 ± 179.40	615.08 ± 132.36	−0.369	0.72
C20:0	8.97 ± 1.87 **	14.02 ± 2.34	4.139	0.002
C18:3n6	4.60 ± 0.81 **	2.96 ± 0.92	−3.292	0.008
C18:3n3	19.17 ± 8.74 ***	114.37 ± 19.21	11.05	<0.001
C20:1	12.34 ± 3.16 ***	29.52 ± 5.82	6.355	<0.001
C20:3n6	25.12 ± 4.34	25.41 ± 13.09	0.051	0.96
C20:3n3	4.69 ± 0.56 **	6.84 ± 1.33	3.644	0.005
C22:1n9	7.01 ± 2.71	8.65 ± 2.41	1.111	0.293
C20:4n6	322.15 ± 46.43	296.27 ± 142.60	−0.423	0.681
C23:0	13.89 ± 2.86 **	22.31 ± 5.76	3.206	0.009
C20:5n3	13.79 ± 3.94 **	127.45 ± 66.48	4.181	0.009
C24:0	16.47 ± 2.98 *	13.79 ± 9.34	2.466	0.033
C24:1	13.05 ± 3.45	13.89 ± 7.02	0.263	0.798
C22:6n3	7.96 ± 2.28 **	40.38 ± 15.30	5.136	0.003
SFA	327.46 ± 95.16	309.18 ± 84.94	−0.351	0.733
MUFA	677.22 ± 223.13	460.77 ± 164.31	−1.913	0.085
PUFA	95.70 ± 20.69	112.32 ± 32.83	1.049	0.319
N3	11.40 ± 1.93 **	72.26 ± 22.30	6.659	0.001
N6	200.11 ± 43.95	187.94 ± 54.41	−0.426	0.679
N6/N3	17.54 ± 2.07 ***	2.62 ± 0.20	−17.58	<0.001

* represents a significant difference (*p* < 0.05); ** represents a very significant difference (*p* < 0.01); *** represents an extremely significant difference (*p* < 0.001); FR: the control group; NR: the dietary garlic skin group; *t*: t-statistic; see Supplementary Table S2 for full data; 1 SFA (saturated fatty acid) = (C4:0 + C6:0 + C8:0 + C10:0 + C11:0 + C12:0 + C13:0 + C14:0 + C15:0 + C16:0 + C17:0 + C18:0 + C20:0 + C21:0 + C22:0 + C23:0 + C24:0); 2 MUFAs (monounsaturated fatty acids) = (C14:1 + C15:1 + C16:1 + C17:1 + C18:1n9t + C18:1n9c + C20:1 + C22:1n9 + C24:1); 3 PUFAs (polyunsaturated fatty acids) = (C18:2n6t + C18:2n6c + C18:3n3 + C18:3n6 + C20:2 + C20:3n6 + C20:3n3 + C20:4n6 + C22:2 + C20:5n3 + C22:6n3); 4 n-3 (omega-3 polyunsaturated fatty acids) = (C18:3n3 + C20:5n3 + C20:3n3 + C22:6n3); 5 n-6 (omega-6 polyunsaturated fatty acids) = (C18:2n6t + C18:2n6c + C18:3n6 + C20:3n6 + C20:4n6).

## Data Availability

Some of the datasets have been uploaded as an attachment to the article, and the datasets used in the current study are available from the corresponding author upon reasonable request.

## Supplementary Materials

The following supporting information can be downloaded at https://www.mdpi.com/article/10.3390/foods14111911/s1: Figure S1. (a) The score plots of OPCA-DA plots of the comparison for the differentially abundant metabolites between the two groups; (b): The permutations test of OPLS-DA in the positive ion detection mode, which can be used for model validity assessment; Table S1: Nutritional composition and bioactive compounds of garlic skin; Table S2: The original data of fatty acid differences between FR and NR; Table S3: The 20 most enriched differential metabolic pathways between FR and NR; Table S4: The top 50 differentially abundant metabolites with the smallest **p**-values between FR and NR.
