# Supplementary material for: Effects of Dietary Garlic Skin Based on Metabolomics Analysis in the Meat Quality of Black Goats
Source: Foods. 2025 May 28;14(11):1911. doi: 10.3390/foods14111911 (PMC12153991; doi:10.3390/foods14111911)
Supplement: Supplementary file 1 [file foods-14-01911-s001.zip › Table S1 .pdf]

Table S1 Nutritional composition and bioactive compounds of garlic skin

Chemical composition, % DM	Contents
Dry matter	90.85
Crude protein	13.55
Ether extracts	4.32
Ash	5.87
Neutral detergent fibre	42.36
Acid detergent fibre	36.57
Bioactive compounds, mg/g	
Total polyphenols content	6.35
Total flavonoid content	0.48
