# Supplementary figures and images for: Effects of Dietary Garlic Skin Based on Metabolomics Analysis in the Meat Quality of Black Goats
Source: Foods. 2025 May 28;14(11):1911. doi: 10.3390/foods14111911 (PMC12153991; doi:10.3390/foods14111911)

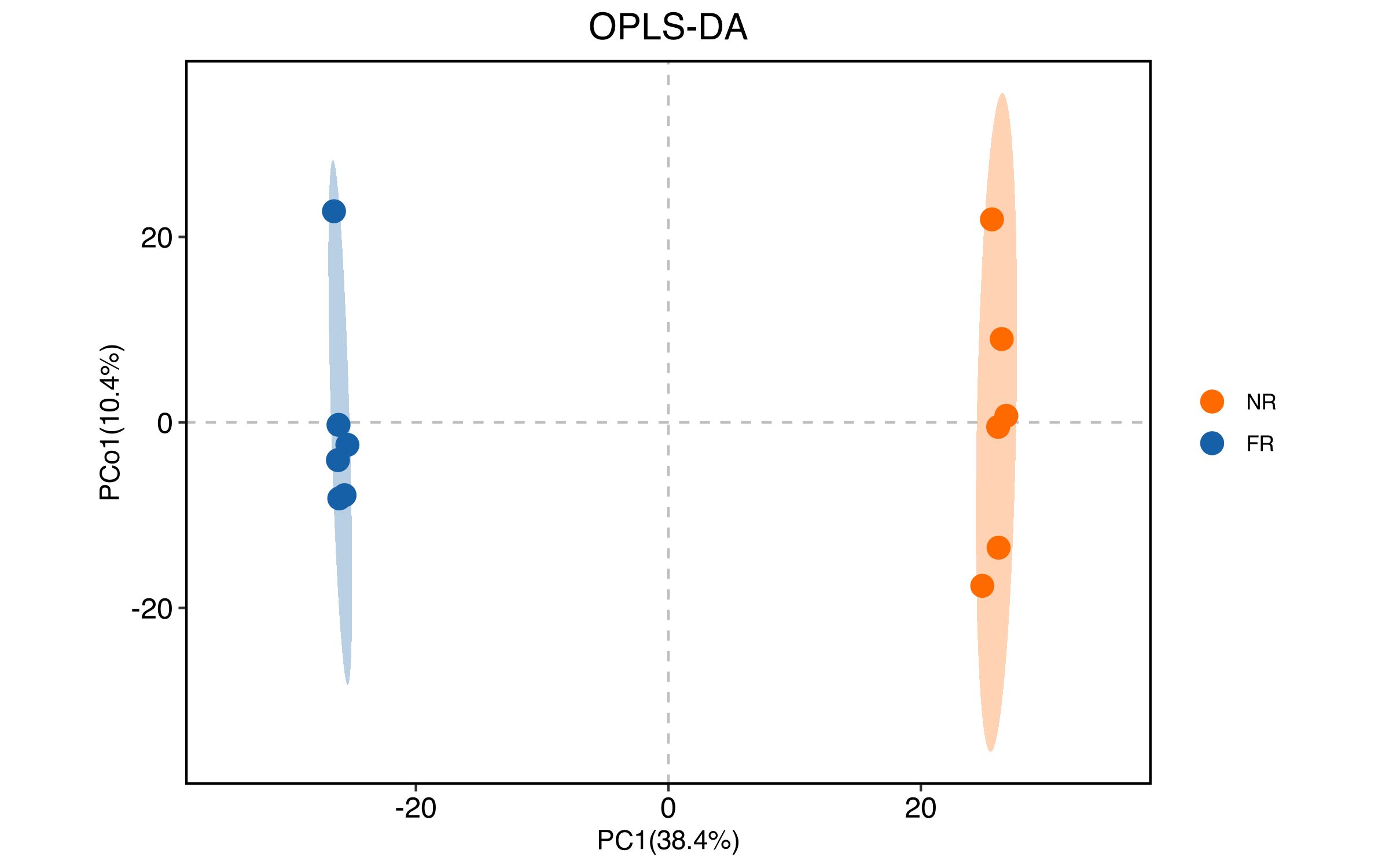

Supplement: Supplementary file 1 [file foods-14-01911-s001.zip › Fig.1a.png]

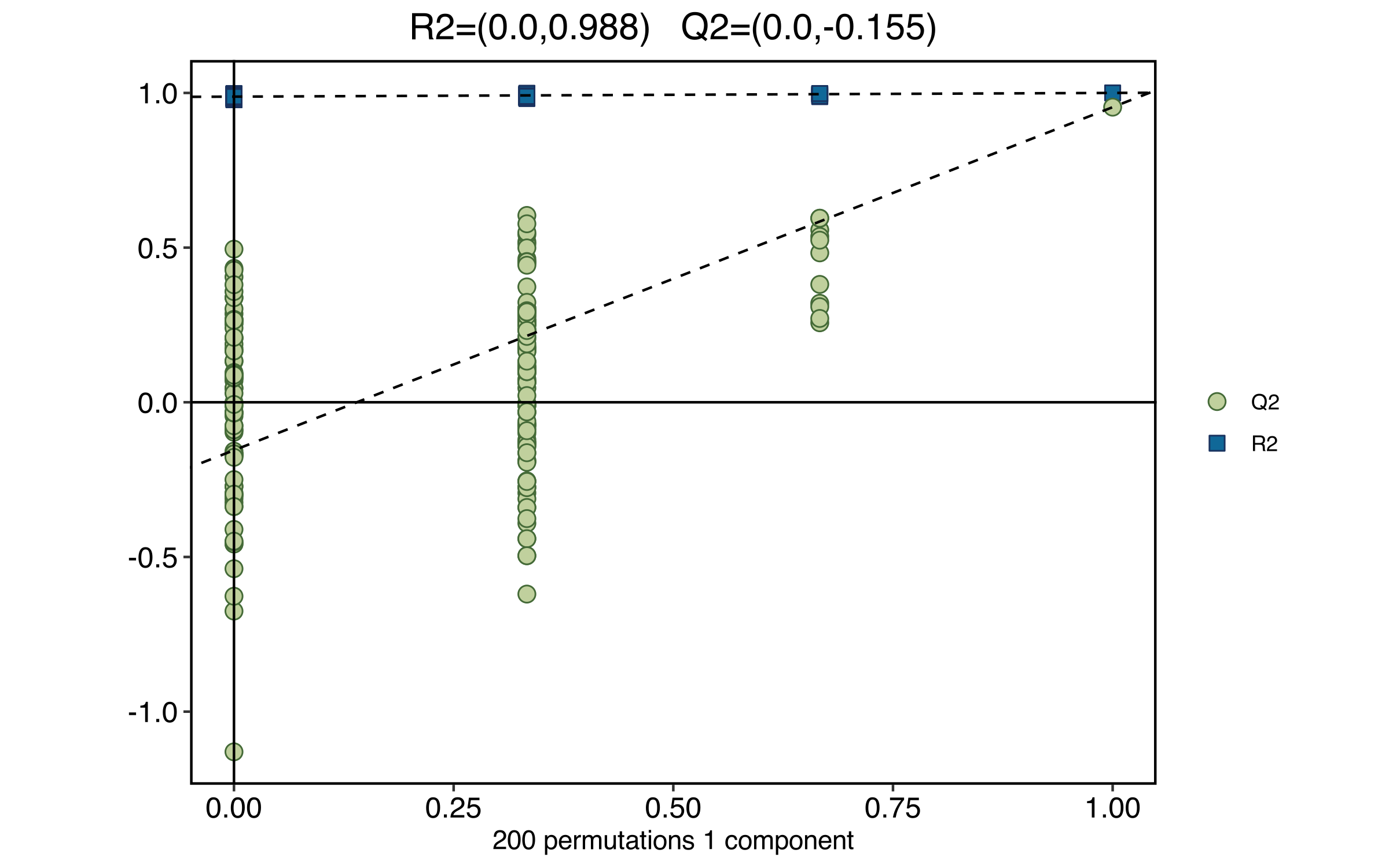

Supplement: Supplementary file 1 [file foods-14-01911-s001.zip › Fig.1b.png]
